# Supplementary material for: Generalizable brain network markers of major depressive disorder across multiple imaging sites
Source: PLoS Biol. 2020 Dec 7;18(12):e3000966. doi: 10.1371/journal.pbio.3000966 (PMC7721148; doi:10.1371/journal.pbio.3000966)
Supplement: S1 Text — (DOCX) [file pbio.3000966.s002.docx]

**S1 Text. Prediction performance using SVM**

Since the performance levels of the prediction models were acceptable, we further tried a support vector machine (SVM) for classification. However, the performance was not improved compared to that in LASSO. In the discovery dataset, the classifier of SVM separated major depressive disorder (MDD) and healthy control (HC) populations with an accuracy of 70%. The corresponding area under the curve (AUC) sensitivity and specificity were 0.73, 61%, and 76%, respectively. This approach led to a Matthews correlation coefficient (MCC) of 0.37. In the independent validation dataset, the classifier of SVM separated the MDD and HC populations with an accuracy of 68%. The corresponding AUC, sensitivity, and specificity were 0.74, 62%, and 73%, respectively. This approach led to an MCC of 0.34.
