# Supplementary material for: Generalizable brain network markers of major depressive disorder across multiple imaging sites
Source: PLoS Biol. 2020 Dec 7;18(12):e3000966. doi: 10.1371/journal.pbio.3000966 (PMC7721148; doi:10.1371/journal.pbio.3000966)
Supplement: S2 Text — (DOCX) [file pbio.3000966.s003.docx]

**S2 Text. Analysis and validation of controls for confound artifact**

The most reliable way to confirm that the classifier's performance is not driven by age is to test if we could obtain adequate generalization performance in age-controlled datasets. Therefore, we prepared 1,000 age matched subsamples from the validation dataset (differences in mean age between major depressive disorder (MDD) and healthy controls (HCs) in the 1,000 subsamples: *t*-value = 0.0052 ± 0.042 [mean ± 1SD], S1 Fig). MDD data were the same but HC data were different among the 1,000 subsamples. The generalization performances were almost the same as those using the whole validation dataset (AUC = 0.756 ± 0.0076 [mean ± 1SD], S1 Fig). AUC, area under the curve.
