# Supplementary material for: Generalizable brain network markers of major depressive disorder across multiple imaging sites
Source: PLoS Biol. 2020 Dec 7;18(12):e3000966. doi: 10.1371/journal.pbio.3000966 (PMC7721148; doi:10.1371/journal.pbio.3000966)
Supplement: S3 Text — (DOCX) [file pbio.3000966.s004.docx]

**S3 Text. Utility of harmonization**

We assessed whether the prediction performance and the number of selected functional connections (FCs) differed depending on the harmonization scheme for the discovery dataset and the independent validation dataset, respectively. We investigated the number of selected FCs because we hypothesized that the harmonization could improve not only the prediction performance but also the information (on important connections for the prediction) that we can extract from the dataset. In total, we constructed 3 brain network markers (without harmonization, with ComBat harmonization, or with traveling subject (TS) harmonization for the discovery dataset) and tested their prediction performances in the independent validation dataset with a ComBat harmonization scheme or without any harmonization. We tested our hypothesis that harmonization improves the degree of separation of the depression probability distributions for healthy control (HC) and major depressive disorder (MDD) groups. A statistic was calculated by *p*(MDD) – 0.5 for MDD and 0.5 – *p*(MDD) for HC. A paired *t*-test was performed to compare the statistics for the individual classifiers. As a result, we found significant improvements in the prediction performance by harmonization for the independent validation dataset (Without vs ComBat: *t_439_* = 4.41, *p* = $1.3\times{10}^{-5}$). In addition, when we estimated the cutoff value for the probability of MDD distribution of the data from OpenNeuro (OTHER), the cutoff value became more plausible (0.59🡪0.54) after harmonization in conjunction with the independent validation dataset (S2 Fig). In contrast, we found no significant improvement in the prediction performance by harmonization for the discovery dataset, finding, instead, significant depreciation (Without vs ComBat: *t_439_* = 0.47, *p* = 0.64), Without vs TS: *t_439_* = -2.50, *p* = 0.013). Although a decrement of the area under the curve value was only 0.01, this result indicates that TS harmonization may worsen the prediction performance. This may be due to the difference in the harmonization method between the discovery dataset (TS harmonization) and the validation dataset (ComBat harmonization). The reason why harmonization for the independent validation dataset improved the prediction performance, but the harmonization for the discovery dataset did not improve the prediction performance, may be due to the fact that the discovery dataset was acquired using a unified imaging protocol. Thus, the effect of harmonization might be small. On the other hand, the number of selected FCs was the largest for TS harmonization (25 FCs), compared to 23 and 21 for without harmonization and ComBat, respectively. This result indicates that we could extract more information on MDD from data with the TS harmonization method.
