# Supplementary material for: Generalizable brain network markers of major depressive disorder across multiple imaging sites
Source: PLoS Biol. 2020 Dec 7;18(12):e3000966. doi: 10.1371/journal.pbio.3000966 (PMC7721148; doi:10.1371/journal.pbio.3000966)
Supplement: S4 Text — (DOCX) [file pbio.3000966.s005.docx]

**S4 Text. Differences in prediction performance across imaging sites**

To investigate whether the prediction performances were different among imaging sites in the independent validation dataset, we calculated the 95% confidence interval (CI) of discrimination performances (area under the curve [AUC], accuracy, sensitivity, and specificity) in every imaging site using a bootstrap method. We repeated the bootstrap procedure 1,000 times and calculated the 95% CI for every site. We then checked whether there is a site whose CI does not overlap with the CIs of other imaging sites. We were not able to find such an imaging site, suggesting no significant systematic difference (S3 Fig).
