# Supplementary material for: Generalizable brain network markers of major depressive disorder across multiple imaging sites
Source: PLoS Biol. 2020 Dec 7;18(12):e3000966. doi: 10.1371/journal.pbio.3000966 (PMC7721148; doi:10.1371/journal.pbio.3000966)
Supplement: S5 Text — (DOCX) [file pbio.3000966.s006.docx]

**S5 Text. Higher/lower resolution of regions of interest**

We assessed the prediction performance when we used a parcellation scheme other than Glasser’s region of interest (ROI). We investigated Shen’s ROI [2] and Power’s ROI [3], which are widely used in previous studies [4, 5]. As a result, the area under the curve (AUC) in the independent validation dataset was 0.74 for Shen’s ROI and 0.76 for Power’s ROI, respectively. This result indicates that there was not much of a difference compared with the prediction performance using Glasser’s ROI (AUC=0.74).

Furthermore, we investigated the effect of a higher/lower resolution in the parcellation on prediction performance using Schaefer’s ROI [6]. Since there are approximately 100 to 1,000 ROIs in increments of 100, Schaefer’s ROI is suitable for investigating the effect of higher/lower resolution parcellations. We excluded 1,000 ROI from this analysis because the scanned volume did not cover all 1,000 ROIs. As a result, the max AUC was achieved when we used 400 ROIs (AUC = 0.75). The AUC increased monotonically up to 400 ROIs, and decreased for over 400 ROIs (S4 Fig). The accuracy as a function of the number of ROI showed a similar profile to the AUC, but the maximum accuracy was achieved when we used 500 ROIs (accuracy = 69%). As such, our choice of Glasser’s 379 ROIs was adequate. AUC, area under the curve.

**References**

1. Kohoutova L, Heo J, Cha S, Lee S, Moon T, Wager TD, et al. Toward a unified framework for interpreting machine-learning models in neuroimaging. Nat Protoc. 2020;15(4):1399-435. doi: 10.1038/s41596-019-0289-5. PubMed PMID: 32203486.

2. Shen X, Tokoglu F, Papademetris X, Constable RT. Groupwise whole-brain parcellation from resting-state fMRI data for network node identification. Neuroimage. 2013;82:403-15. doi: 10.1016/j.neuroimage.2013.05.081. PubMed PMID: 23747961; PubMed Central PMCID: PMCPMC3759540.

3. Power JD, Cohen AL, Nelson SM, Wig GS, Barnes KA, Church JA, et al. Functional network organization of the human brain. Neuron. 2011;72(4):665-78. doi: 10.1016/j.neuron.2011.09.006. PubMed PMID: 22099467; PubMed Central PMCID: PMCPMC3222858.

4. Drysdale AT, Grosenick L, Downar J, Dunlop K, Mansouri F, Meng Y, et al. Resting-state connectivity biomarkers define neurophysiological subtypes of depression. Nat Med. 2017;23(1):28-38. doi: 10.1038/nm.4246. PubMed PMID: 27918562; PubMed Central PMCID: PMCPMC5624035.

5. Rosenberg MD, Finn ES, Scheinost D, Papademetris X, Shen X, Constable RT, et al. A neuromarker of sustained attention from whole-brain functional connectivity. Nat Neurosci. 2016;19(1):165-71. doi: 10.1038/nn.4179. PubMed PMID: 26595653; PubMed Central PMCID: PMCPMC4696892.

6. Schaefer A, Kong R, Gordon EM, Laumann TO, Zuo XN, Holmes AJ, et al. Local-Global Parcellation of the Human Cerebral Cortex from Intrinsic Functional Connectivity MRI. Cereb Cortex. 2018;28(9):3095-114. doi: 10.1093/cercor/bhx179. PubMed PMID: 28981612; PubMed Central PMCID: PMCPMC6095216.
