# Supplementary material for: Generalizable brain network markers of major depressive disorder across multiple imaging sites
Source: PLoS Biol. 2020 Dec 7;18(12):e3000966. doi: 10.1371/journal.pbio.3000966 (PMC7721148; doi:10.1371/journal.pbio.3000966)
Supplement: S6 Text — (DOCX) [file pbio.3000966.s007.docx]

**S6 Text. Generalization of the classifiers to other disorders.**

In addition to a brain network marker of MDD, we developed brain network markers of schizophrenia (SCZ) and autism spectrum disorder (ASD) using the same method as in the main text. We sought to investigate and confirm the spectral structure among the disorders as revealed by previous studies [1-3]. For example, if the MDD classifier predicts patients with a different disorder as patients with MDD, then the probability of diagnosis for patients with that disorder should be over 0.5. In this case, we may say that the patients possess some degree of MDD-ness and that this disorder is related to MDD according to the imaging biological dimension.

Specifically, we first developed SCZ and ASD markers that distinguished between HCs and patients. We used 564 HCs from the discovery dataset in the main text, 102 patients with SCZ from 3 sites, and 121 patients with ASD from 2 sites (S6 Table). Data were acquired using the same protocols as for the discovery dataset. We tested the generalizability of the SCZ marker using an independent validation dataset for patients with SCZ (52 patients with SCZ and 75 HCs from one site, S4 Table). Since we did not have an independent validation dataset for patients with ASD, we tested the performance of the ASD marker using the 10-fold CV. We achieved acceptable performance for both the SCZ marker (Discovery dataset: AUC = 0.85, accuracy = 78%, sensitivity = 75%, specificity = 79%, Independent validation dataset: AUC = 0.87, accuracy = 78%, sensitivity = 81%, specificity = 77%) and ASD marker (Discovery dataset: AUC = 0.76, accuracy = 65%, sensitivity = 73%, specificity = 63%). We then applied these brain network markers to patients with other disorders. We computed the probability of diagnosis in the MDD classifier, that is, the MDD-ness of individual patients within the SCZ and ASD data, and vice versa (S5 Fig).

As a result, we found that patients with SCZ have high MDD-ness (accuracy = 74%, *p* = $7.4\times{10}^{-7}$, two-way binomial test) and ASD-ness (accuracy = 62%, *p* = 0.013, two-way binomial test). In contrast, patients with MDD did not have high SCZ-ness (accuracy = 46%, *p* = 0.35, two-way binomial test) or ASD-ness (accuracy = 54%, *p* = 0.27, two-way binomial test), and patients with ASD did not have high SCZ-ness (accuracy = 42%, *p* = 0.10, two-way binomial test) or MDD-ness (accuracy = 53%, *p* = 0.47, two-way binomial test). AUC, area under the curve.

**References**

1. Ichikawa N, Lisi G, Yahata N, Okada G, Takamura M, Hashimoto RI, et al. Primary functional brain connections associated with melancholic major depressive disorder and modulation by antidepressants. Scientific reports. 2020;10(1):3542. doi: 10.1038/s41598-020-60527-z. PubMed PMID: 32103088; PubMed Central PMCID: PMCPMC7044159.

2. Yahata N, Morimoto J, Hashimoto R, Lisi G, Shibata K, Kawakubo Y, et al. A small number of abnormal brain connections predicts adult autism spectrum disorder. Nat Commun. 2016;7:11254. Epub 2016/04/15. doi: 10.1038/ncomms11254. PubMed PMID: 27075704; PubMed Central PMCID: PMCPMC4834637.

3. Yoshihara Y, Lisi G, Yahata N, Fujino J, Matsumoto Y, Miyata J, et al. Overlapping but Asymmetrical Relationships Between Schizophrenia and Autism Revealed by Brain Connectivity. Schizophr Bull. 2020. doi: 10.1093/schbul/sbaa021. PubMed PMID: 32300809.
