# Supplementary figures and images for: Generalizable brain network markers of major depressive disorder across multiple imaging sites
Source: PLoS Biol. 2020 Dec 7;18(12):e3000966. doi: 10.1371/journal.pbio.3000966 (PMC7721148; doi:10.1371/journal.pbio.3000966)

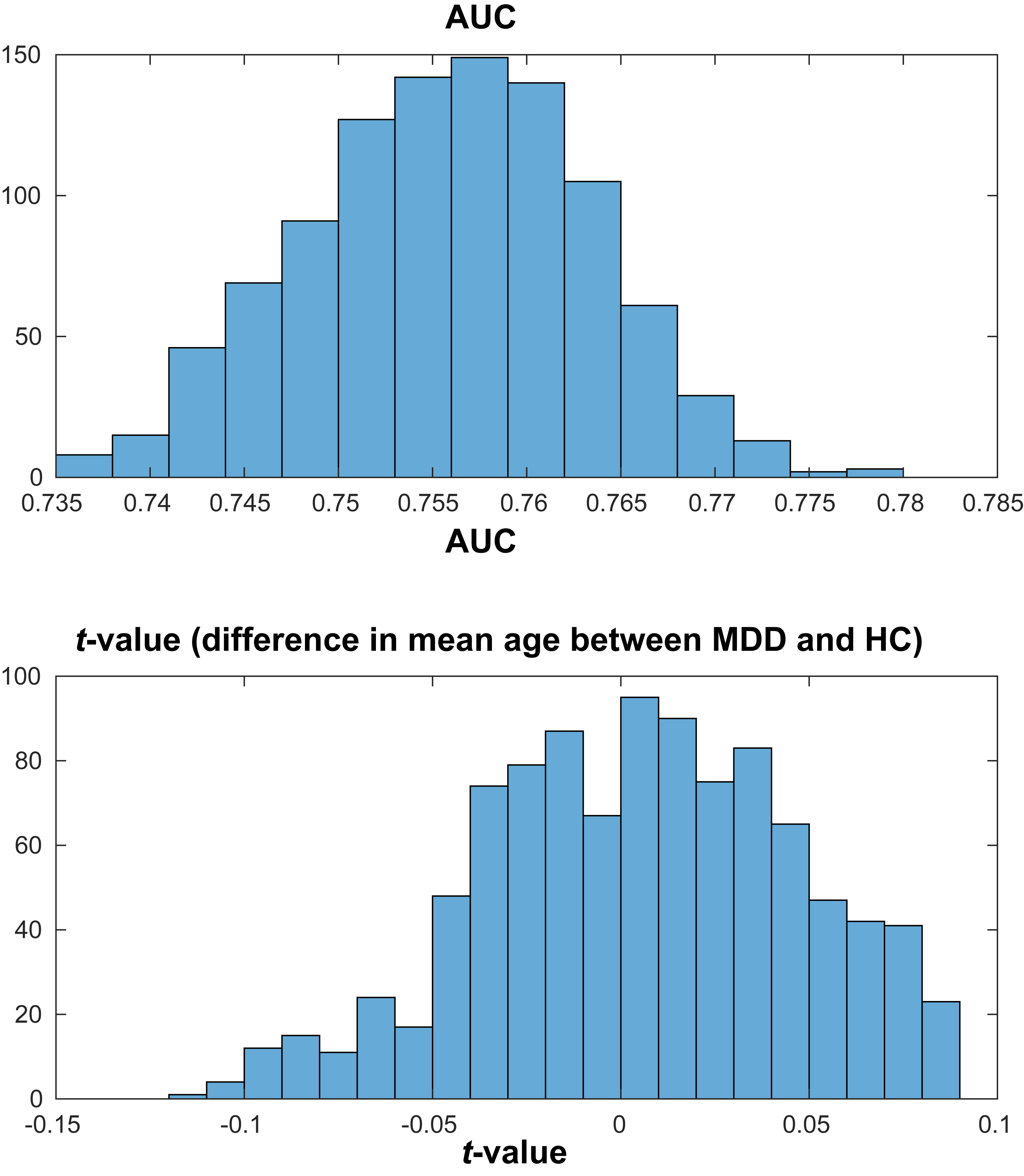

Supplement: S1 Fig — The distribution of the AUC and t-value (difference in mean age between MDD and HC groups) across all subsamples. The numerical data used in this figure are included in S1 Data. AUC, area under the curve; HC, healthy control; MDD, major depressive disorder. (TIF) [file pbio.3000966.s008.tif]

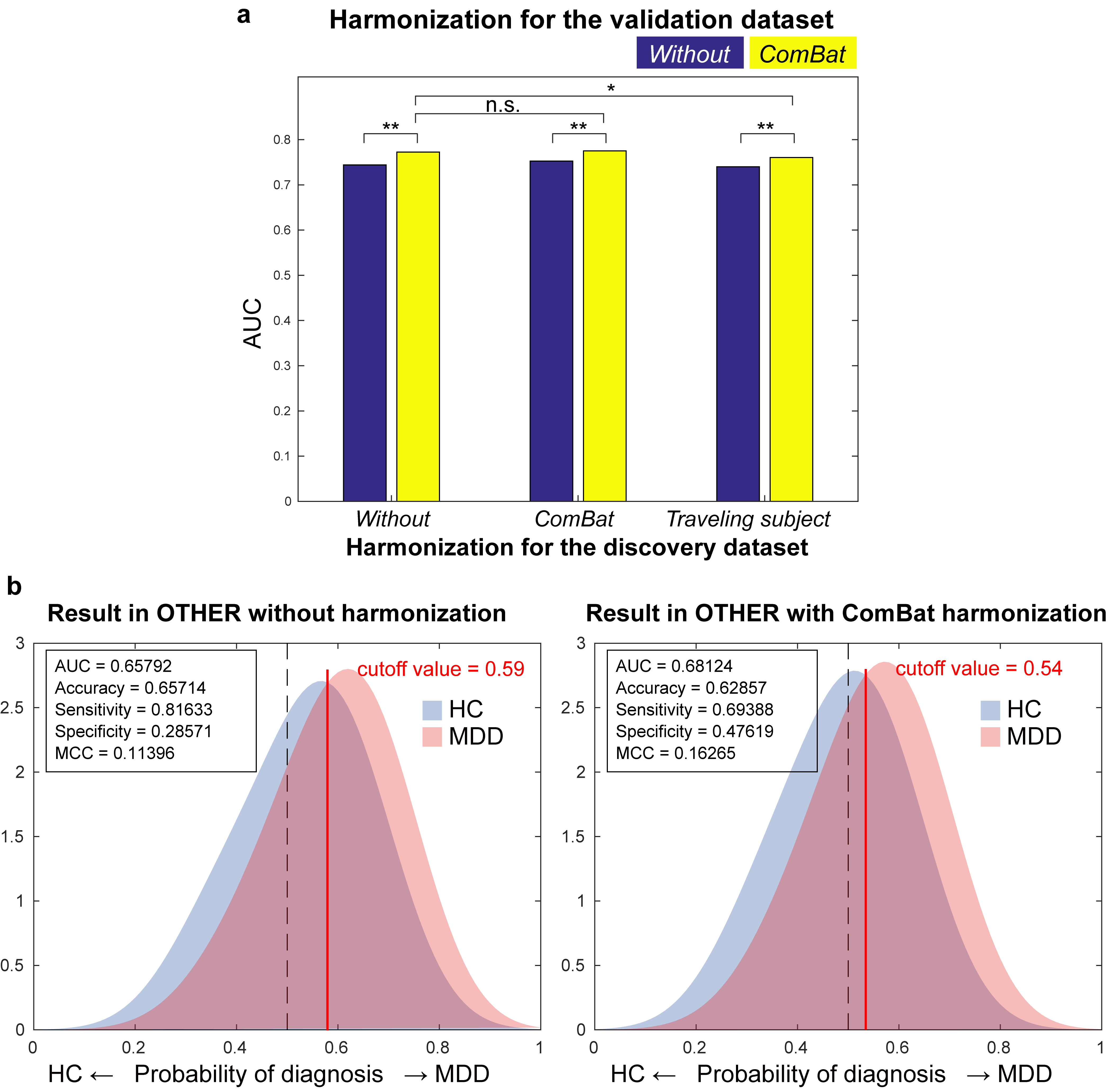

Supplement: S2 Fig — (a) The prediction performance (AUC) of the MDD classifier in the independent validation dataset for each harmonization scheme for the discovery dataset and the independent validation dataset (without harmonization; blue bar, ComBat harmonization; yellow bar). (b) Probability distributions for the diagnosis of MDD in the data from OpenNeuro (OTHER) without harmonization or with ComBat harmonization. The numerical data used in this figure are included in S1 Data. AUC, area under the curve; HC, healthy control; MCC, Matthews correlation coefficient; MDD, major depressive disorder. (TIF) [file pbio.3000966.s009.tif]

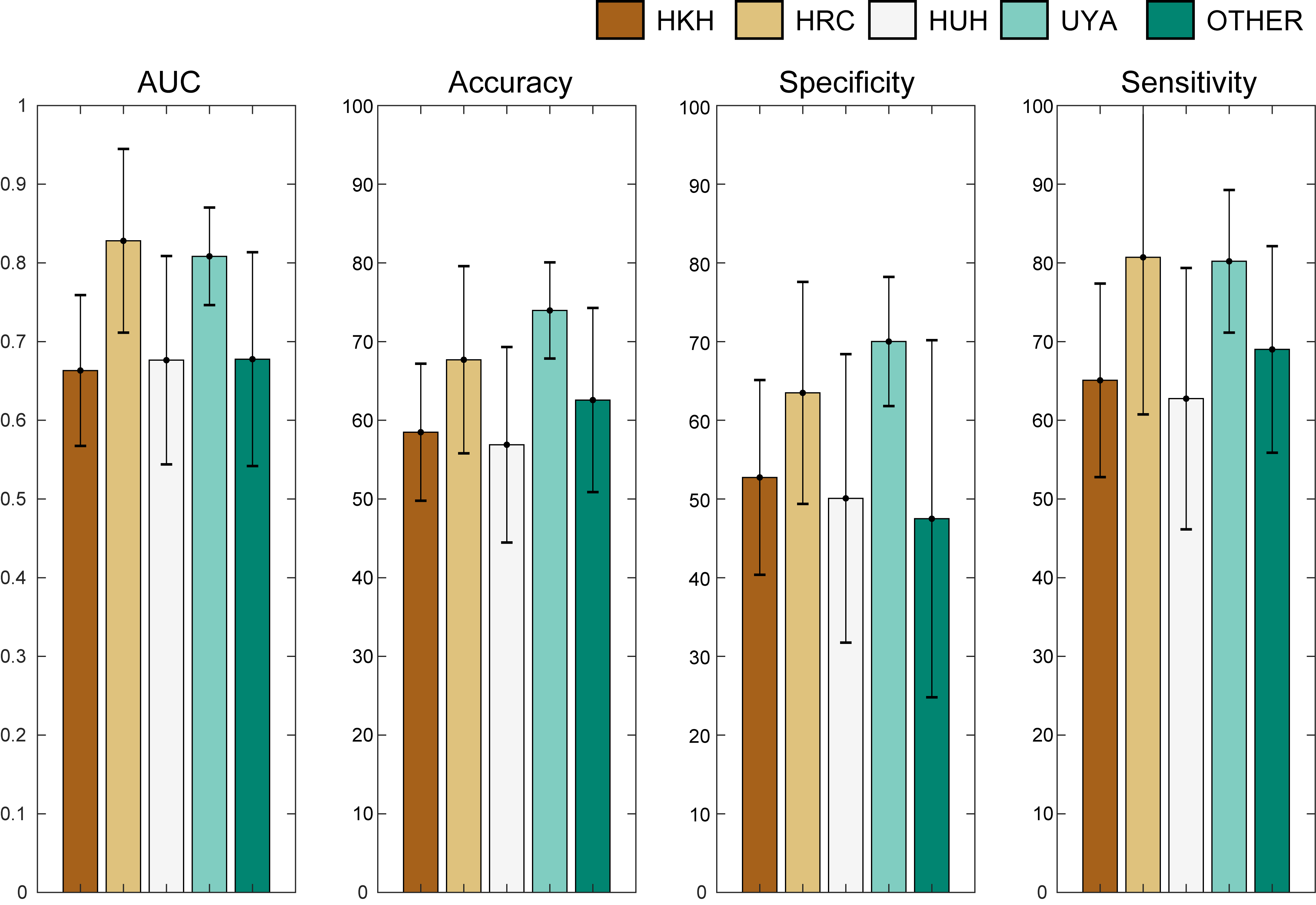

Supplement: S3 Fig — Prediction performances of the MDD classifier in the independent validation dataset in each site. Each color bar indicates a site. Error bar shows the 95% confidence interval from the bootstrap. The numerical data used in this figure are included in S1 Data. AUC, area under the curve; HKH, Hiroshima Kajikawa Hospital; HRC, Hiroshima Rehabilitation Center; HUH, Hiroshima University Hospital; MDD, major depressive disorder; UYA, Yamaguchi University. (TIF) [file pbio.3000966.s010.tif]

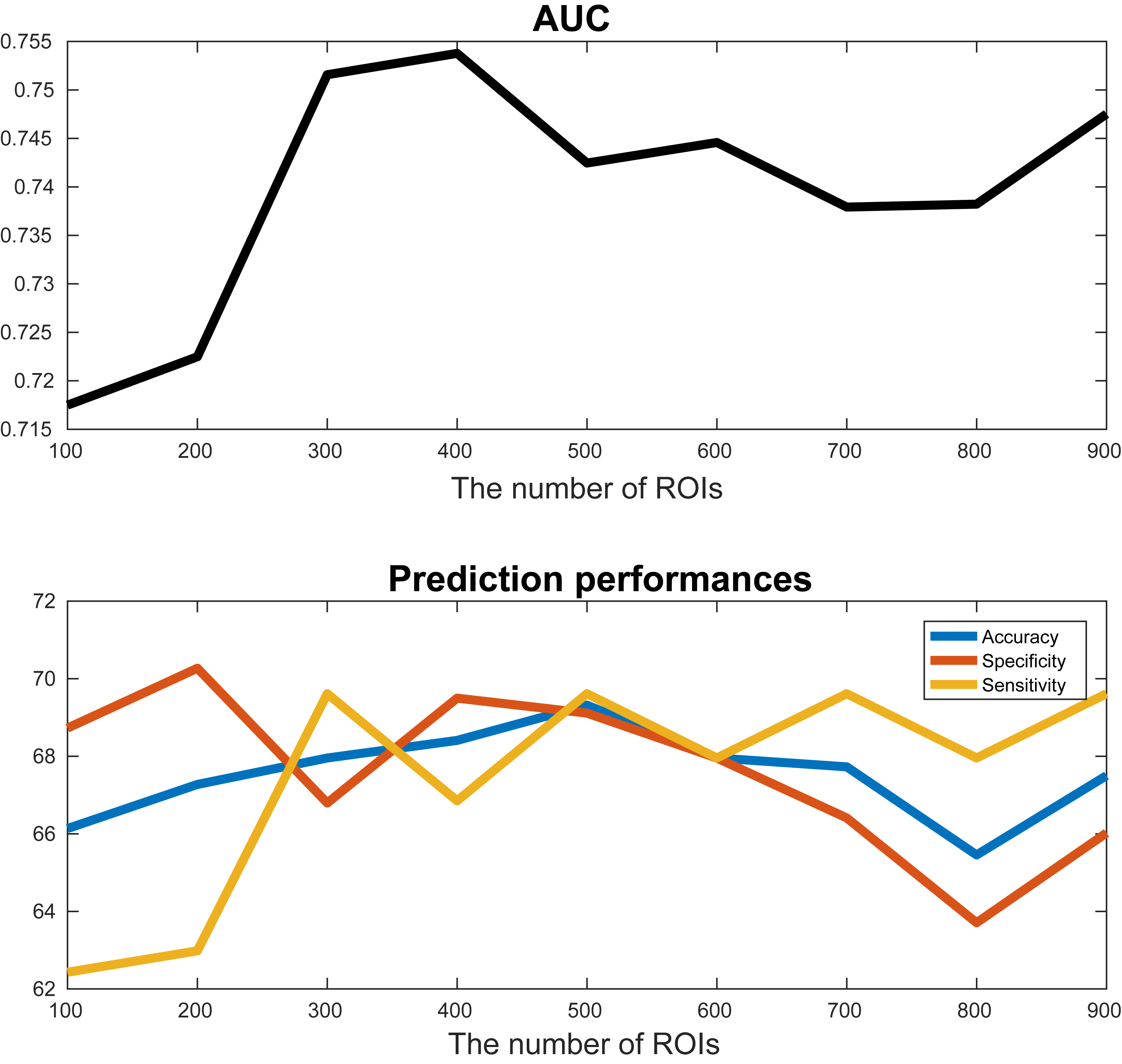

Supplement: S4 Fig — The prediction performances (AUC, accuracy, specificity, and sensitivity) of the MDD classifier constructed by Schaefer’s ROIs as a function of the number of ROIs. The numerical data used in this figure are included in S1 Data. AUC, area under the curve; MDD, major depressive disorder; ROI, region of interest. (TIF) [file pbio.3000966.s011.tif]

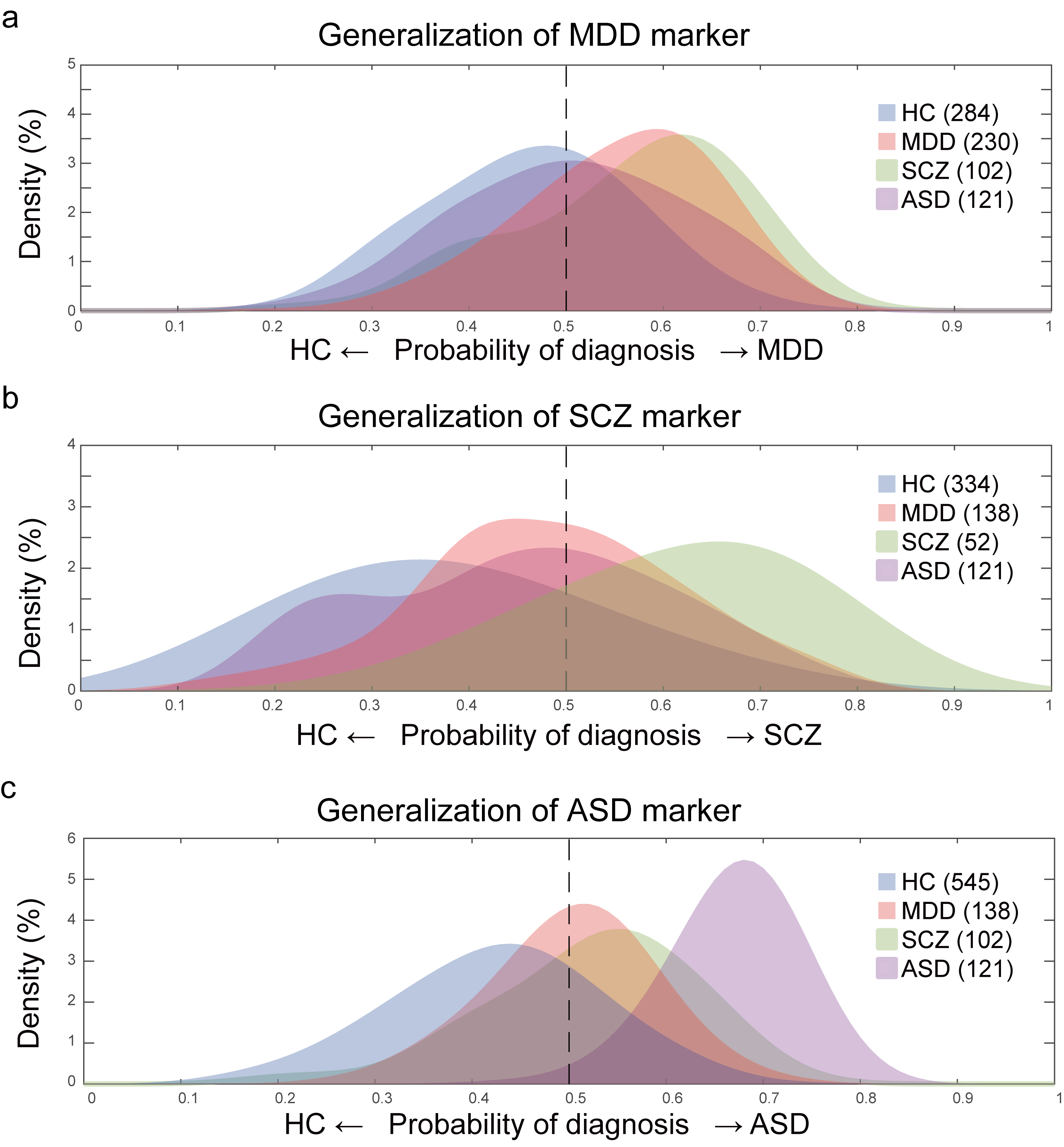

Supplement: S5 Fig — Density distributions of the probability of diagnosis obtained by applying (a) the MDD marker, (b) SCZ marker, and (c) ASD marker to the HCs and patients with MDD, SCZ, and ASD. In each panel, the patient distribution and the HC distribution are plotted separately, with the colored areas representing one or the other. The numbers in parentheses next to HC, MDD, ASD, and SCZ in each panel indicate the number of subjects in the distributions. The independent validation dataset was used in a and b. HCs in a, b, and c were scanned at the same sites as their corresponding patient data. The numerical data used in this figure are included in S1 Data. ASD, autism spectrum disorder; HC, healthy control; MDD, major depressive disorder; SCZ, schizophrenia. (TIF) [file pbio.3000966.s012.tif]
