# Supplementary material for: Generalizable brain network markers of major depressive disorder across multiple imaging sites
Source: PLoS Biol. 2020 Dec 7;18(12):e3000966. doi: 10.1371/journal.pbio.3000966 (PMC7721148; doi:10.1371/journal.pbio.3000966)
Supplement: Attachment — Submitted filename: ResponseLetter_PLOSBiology_R1.docx [file pbio.3000966.s019.docx]

**Response to Academic Editor:**

We appreciate the editor’s critical comments and helpful suggestions. We have taken all the comments and suggestions into account, which helped us to improve our manuscript. Our responses to the editor’s comments are below.

***Editor’s comment 1:*** *“1) how can harmonization be applied to independent test cohorts without information leakage? What would be the procedure for applying this data prospectively to new cohorts?”*

***Response:*** Thank you very much for this insightful comment. In this paper, information leakage does not occur because harmonization was performed separately for the discovery dataset and validation dataset. We used this procedure because we anticipate its clinical application. In our current procedure, it is important to harmonize the data among imaging sites within the validation dataset. Therefore, we needed the validation dataset collected from multiple imaging sites. However, in the future, we will seek to investigate how many imaging sites are needed for harmonization. Besides, as an alternative, we could achieve harmonization without information leakage by harmonizing the average values of every functional connectivity in the new data to those in the data used for the model construction.

For the clinical application of our harmonization method to actual medical practice, the best classification performance will be achieved when 1) all imaging sites are involved in a traveling subject dataset, 2) the traveling subject harmonization is applied throughout the discovery and the validation datasets, and 3) a brain network marker is reconstructed from the harmonized datasets. However, when we use this method under a medical device program approval, it seems more realistic to conduct harmonization without using the discovery dataset because any imaging sites cannot be added to the dataset after the approval. In other words, the method in this paper or the method that applying the traveling subject harmonization within the independent validation dataset should be preferred. It is important to note that due to the absence of the traveling subject dataset, traveling subject harmonization was not possible for the independent validation dataset, and we were forced to use ComBat in this case. We added this explanation to the Discussion section.

***Changes:*** The Discussion section of the manuscript now contains the following paragraph: “For the application of our harmonization method to the actual medical field,…” (page 22 lines 481–491).

***Editor’s comment 2:*** *“2) can you rule out the possibility that the classifier's performance is driven by confounds, particularly head movement? One approach is to test whether classifier scores (i.e., probability of depression) can be predicted from combinations of head movement parameters (e.g., as in Kohoutova et al. 2020, Nat. Protocols).*”

***Response:*** We agree with the editor’s comment that it is important to rule out the possibility that the classifier’s performance is driven by confounds. Therefore, we tested whether we could predict classifier scores (probability of depression) based on age, sex, amount of motion (frame-wise displacement [FD]), or the combination of head movement parameters (x, y, z, yaw, pitch, and roll) (Kohoutova et al., 2020), respectively. We evaluated the performances by a 10-fold cross-validation in the discovery dataset. As a result, only age could significantly predict the classifier scores (age: *r* = 0.26, *p* < 0.0001, sex: *r* = 0.069, *p* > 0.07, FD: *r* = -0.031, *p* > 0.42, motion parameters: *r* = 0.06, *p* > 0.09). This result indicates that the classifier’s performance was influenced by age, but not sex, amount of motion, or the combination of head movement parameters.

Given the above, to remove the age effect on the classifier’s scores, we changed the method for constructing a classifier. When we performed an undersampling and subsampling procedure, we matched the mean age between major depressive disorder (MDD) and healthy control (HC) groups in each subsample. As a result, the area under the curve (AUC), accuracy, sensitivity, and specificity were 0.74, 66%, 72%, and 65%, respectively, in the discovery dataset. In the validation dataset, AUC, accuracy, sensitivity, and specificity were 0.76, 68%, 70%, and 66%, respectively. These results indicate that the classifier performances did not change even after changing the method. Next, we tested whether we can predict classifier scores from age by a 10-fold cross-validation. Although only age could predict the classifier scores (age: *r* = 0.14, *p* < 0.0005, sex: *r* = 0.029, *p* > 0.45, FD: *r* = 0.07, *p* > 0.05, motion parameters: *r* = 0.038, *p* > 0.32), the effect decreased. This result raises two possibilities: 1) the classifier outputs were really affected by age, or 2) differences in mean age between MDD and HC groups (mean age: MDD = 41.8, HC = 37.4) led to the pseudo correlation between age and probability of depression. To test the latter possibility, we prepared 10 subsample datasets in which the mean age was matched between MDD and HC groups from the discovery dataset (differences in mean age between MDD and HC in 10 subsamples: *t*-value = -0.021±0.057 [mean ±1 SD]). MDD data were the same but the HC data were different among the 10 subsamples. If the latter is correct, we cannot predict the classifier scores based on age. As a result, we could not significantly predict the classifier scores in any of the subsamples (*r* = 0.0195±0.0677 [mean ± 1SD], *p* = 0.06~0.63). This result suggests that the difference in mean age between MDD and HC groups led to the pseudo correlation. In other words, the classifier's performance was unlikely to have been driven by age. We included all above analyses in the Results section and modified the Methods and Results section to update the method for constructing a classifier, classification performances, and the important functional connections for an MDD diagnosis.

Furthermore, the most reliable way to confirm that the classifier's performance is not driven by age is to test whether we can obtain an adequate generalization performance in age-controlled datasets. Therefore, we prepared 1,000 age matched subsamples from the validation dataset (differences in mean age between MDD and HC in the 1,000 subsamples: *t*-value = 0.0052 ± 0.042 [mean ± 1SD], S1 Fig). MDD data were the same but HC data were different across the 1,000 subsamples. The generalization performances were almost the same as those using the whole validation dataset (AUC = 0.756 ± 0.0076 [mean ± 1SD], S1 Fig). In addition to this result, our new result demonstrating our success in the generalization (AUC = 0.68) to the dataset collected from a country outside Japan (OpenNeuro: <https://openneuro.org/datasets/ds002748/versions/1.0.0>) also ruled out the possibility that the classifier’s performance was driven by age, because the mean age was matched between MDD and HC groups in this dataset (MDD = 32.7 ± 8.94 [mean ± 1SD], HC = 33.8 ± 8.49 [mean ± 1SD], *t*-test: *t*_68_ = -0.5, *p* = 0.62) (see detail in our response to the reviewer 3’s comment 3). We added this analysis to the S2 Text and S1 Figure in the supplementary information.

***Changes:*** Following the change in the method to construct a brain network marker, we changed all main results corresponding to the change in the method (prediction performances and selected functional connections).

We have revised the Results section of the manuscript, which now contains the following paragraph: “To rule out the possibility that the classifier’s performance is…” (page 15 line 313–page 16 line 320).

**References:**

Kohoutova, L., Heo, J., Cha, S., Lee, S., Moon, T., Wager, T. D., & Woo, C. W. (2020). Toward a unified framework for interpreting machine-learning models in neuroimaging. Nat Protoc, 15(4), 1399-1435. doi:10.1038/s41596-019-0289-5

**S1 Fig. Distribution of prediction performance and difference in mean age between MDD and HC across all subsamples.** The distribution of area under the curve (AUC) and *t*-value (difference in mean age between major depressive disorder (MDD) and healthy control (HC) groups) across all subsamples.

**Response to Reviewer #1:**

We appreciate the reviewer’s critical comments and helpful suggestions. We have taken all the comments and suggestions into account, which helped us to improve our manuscript. Our responses to the reviewer’s comments are below.

***Reviewer’s comment 1:*** *“A main point is that it is unclear as to how significant the 69% classification accuracy actually is for differentiating between MDD patients and healthy controls in clinical practice, given the relative ease at distinguishing patients with MDD from healthy controls clinically. A potentially more useful classification would be to identify different subgroups/ MDDD dimensions.”*

***Response:*** While the reviewer’s point is well taken, we believe that a diagnosis of major depressive disorder (MDD) based on DSM is not easy, as a previous study shows a low inter-rater agreement (kappa = 0.28) (Freedman et al., 2013; Regier et al., 2013). In this study, we have developed a brain network marker which can objectively predict diagnosis based on structured interviews, which have a relatively high inter-rater agreement, such as SCID (kappa = 0.66) (Shankman et al., 2018; Williams et al., 1992) or MINI (high agreement with SCID: kappa = 0.85) (Otsubo et al., 2005). Therefore, we believe that it is valuable to objectively predict a diagnosis from relatively low-cost resting-state fMRI data that can be obtained within about 10 minutes.

To objectively compare and verify the stability of a diagnosis by our brain network marker with that of diagnosis by doctors based on the DSM, we performed a new simulation. In this simulation, we prepared a surrogate set of 1,000 participants who have a “true” probability of depression (0 < *p*(MDD) < 1). In reality, we used the classifier’s outputs as the true probability. We assumed that the rater’s noise (*N*(0, Σ)) was added to the probability of depression ($\hat{p}(MDD)$), and that a participant with $\hat{p}(MDD)$ = 0.5 or more is diagnosed with MDD. By assuming two raters, we can calculate a diagnostic agreement rate between these two raters’ diagnosis (kappa). Based on this simulation, we estimated the rater’s variance Σ when kappa was 0.28 (Freedman et al., 2013; Regier et al., 2013), and the Σ was 0.0149.

Next, we evaluated the variance of our classifier’s output across imaging sites. We applied our classifier to a traveling subject dataset in which each of 9 healthy participants was scanned about 50 times at 12 different sites, producing a total of 411 scans (S5 Table). We evaluated the inter-rater variance of our classifier’s output assuming that each MRI scanner is a rater. We estimated whole-variance (variance across all about 50 scans), between-sites-variance (we calculated the average of two or three scans per imaging site and then took the variance across the average values), and within-site-variance (variance across 15 scans at one imaging site) in each participant. As a result, the whole-variance was 0.0061, the between-sites-variance was 0.0037, and the within-site-variance was 0.0055, averaged across participants. These values represented about 25~40% of the variance between raters in the above simulation. These results indicate that the diagnosis by our brain network marker could be more objective and stable than the diagnosis by doctors, even considering the variance across fMRI scanners and imaging sites. We explained these motivation and results in the Introduction, the Results and the Discussion sections.

As the reviewer mentioned, since MDD is a highly heterogeneous condition, our final goal is the redefinition and subtyping of psychiatric disorders, agnostic to diagnosis. Although our hierarchical supervised / unsupervised approach starts with supervised learning based on a diagnosis, a diagnosis is only used as a single piece of information to extract important functional connectivity. We then used the identified functional connections for biologically meaningful dimensions of the disorder and applied unsupervised learning to these low biological dimensions to further understand psychiatric disorders (redefinition and subtyping). To reach the final goal, it is an absolutely necessary prerequisite to first treat MDD as a single entity using diagnoses as supervised data and create a brain network marker that generalizes to a completely independent data collected from multiple imaging sites. We included the above explanation in the Introduction section with greater clarity.

***Changes:*** We have revised the Introduction section of the manuscript, which now contains the following paragraph and sentences: “We believe that diagnosis of MDD based on Diagnostic and Statistical Manual of Mental Disorders (DSM) is not easy…” (page 7 line 146–page 8 line 155), “Major depressive disorder (MDD) is a highly heterogeneous psychiatric disorder, …” (page 4 lines 55–75), “Our purpose at this stage is to find …” (page 5 lines 80–83), “a lumping category such as a diagnosis,…” (page 5 lines 90–91), and “To achieve the final goal (redefinition and subtyping),…” (page 6 lines 105–108).

We have revised the Results section of the manuscript, which now contains the following paragraph: “To objectively compare and verify the stability of the diagnoses…” (page 17 lines 344–362).

We have revised the Discussion section of the manuscript, which now contains the following sentence: “Furthermore, our simulation result indicates…” (page 22 lines 478–480).

***References:***

Freedman, R., Lewis, D. A., Michels, R., Pine, D. S., Schultz, S. K., Tamminga, C. A., . . . Oquendo, M. A. (2013). The initial field trials of DSM-5: new blooms and old thorns: Am Psychiatric Assoc.

Otsubo, T., Tanaka, K., Koda, R., Shinoda, J., Sano, N., Tanaka, S., . . . Kamijima, K. (2005). Reliability and validity of Japanese version of the Mini‐International Neuropsychiatric Interview. Psychiatry and clinical neurosciences, 59(5), 517-526.

Regier, D. A., Narrow, W. E., Clarke, D. E., Kraemer, H. C., Kuramoto, S. J., Kuhl, E. A., & Kupfer, D. J. (2013). DSM-5 field trials in the United States and Canada, Part II: test-retest reliability of selected categorical diagnoses. American journal of psychiatry, 170(1), 59-70.

Shankman, S. A., Funkhouser, C. J., Klein, D. N., Davila, J., Lerner, D., & Hee, D. (2018). Reliability and validity of severity dimensions of psychopathology assessed using the Structured Clinical Interview for DSM-5 (SCID). Int J Methods Psychiatr Res, 27(1). doi:10.1002/mpr.1590

Williams, J. B., Gibbon, M., First, M. B., Spitzer, R. L., Davies, M., Borus, J., . . . Rounsaville, B. (1992). The structured clinical interview for DSM-III-R (SCID): II. Multisite test-retest reliability. Archives of general psychiatry, 49(8), 630-636.

***Reviewer’s comment 2:*** *“It is not clear how systematically patient medication status was considered in sensitivity analyses examining the generalizability of the classifier to milder versus more severe depression (p.9).”*

***Response:*** We thank the reviewer for pointing out this important point. We had not systematically considered patient medication status in milder (BDI <= 20) and more severe depression (BDI > 20). We as such assessed whether the proportion of antidepressants, anxiolytics, antipsychotics, and mood stabilizers used in the milder group and more severe group were statistically different. As a result, we found that the milder group used antidepressant significantly less than the severe group (**antidepressant: milder = 19/30, severe =124/155, *z* = 2.00, *p* = 0.046**, anxiolytic: milder = 10/30, severe =65/155, *z* = 0.88, *p* > 0.38, antipsychotic: milder = 9/30, severe =33/155, *z* = 1.04, *p* > 0.3, mood stabilizer: milder = 1/30, severe =13/155, *z* = 0.96, *p* > 0.34). However, since we confirmed there was no difference in the classification accuracy between the two groups (70% and 68% for milder and severe groups, respectively), these results suggest that our brain network marker is not based on the effects of antidepressants on brain circuits. We included this result in the Results section.

***Changes:*** We have revised the Results section of the manuscript, which now contains the following sentences: “In addition, we assessed whether the proportion of antidepressants,…” (page 14 lines 278–286).

***Reviewer’s comment 3:*** *“Was the undersampling procedure in the discovery dataset analyses adequate to control for the fact that the number of patients with MDD was 4 x smaller than that of healthy controls?”*

***Respons*e:** Following the reviewer’s suggestions, we investigated a performance of a brain network marker constructed without the under-sampling procedure (used only 10-fold cross validation). The brain network marker without the under-sampling predicted all participants, including those with MDD, as healthy controls (specificity = 100%, sensitivity = 0%). This result indicates that the under-sampling procedure was both essential and sufficiently effective.

***Reviewer’s comment 4:*** *“There is no description of the nature of the main FC profiles important for classifying MDD in the Results. Similarly, it is only In the Discussion that the authors note findings regarding schizophrenia and ASD patient analyses. It would be helpful to have brief summary of these important findings in the Results section.”*

***Response:*** Following the reviewer’s suggestions, we included the results regarding schizophrenia and autism spectrum disorder analyses in the Results section. Furthermore, we described the nature of the main functional connectivity (FC) profiles in the Results section. Specifically, there are four points as listed below.

1) The FC between the left and right insula revealed the largest difference between patients with MDD and HCs (FC#12 in Fig. 4b).

2) 19 of 25 FCs exhibited “under-connectivity” and only 6 FCs exhibited “over-connectivity.” Note that the state of FC exhibiting the smaller (that is, more negative) and greater (more positive) mean correlation value in the major depressive disorder population than the healthy control population is termed under- and over-connectivity, respectively.

3) 2 FCs (FC#11 and FC#23 in Fig. 4b) were related to the subgenual anterior cingulate cortex (sgACC) which is known as an important treatment target of deep brain stimulation (DBS) for MDD.

4) FC#2 was FC between the sensory motor cortex (postcentral cortex) and the left dorsolateral prefrontal cortex (left DLPFC) which is known as an important treatment target of repetitive transcranial magnetic stimulation (rTMS).

We have modified Figure 4 to summarize the details to better understand and explain the selected FCs in the Discussion section.

***Changes:*** We have revised the Results section of the manuscript, which now contains the following sentences: “We hereafter summarize the characteristics of these FCs…” (page 19 lines 402–409). We have also revised the corresponding part in the Discussion section (page 23 lines 492–509).

**Figure 4: Important FCs for MDD diagnosis. (a)** The 25 functional connections (FCs) viewed from left, back, right, and top. Interhemispheric connections are shown in the back and top views only. Regions are color-coded according to the intrinsic network. The state of functional connectivity exhibiting the smaller (that is, more negative) and greater (more positive) mean correlation index in the major depressive disorder (MDD) population than the healthy control (HC) population is termed under- (blue line) and over-connectivity (red line), respectively. The width of the line represents the effect size of the difference (*t*-value) in the FC values between MDD and HC groups. **(b)** Listed here are the laterality and anatomical identification of the region of interest (ROI) as identified by the Anatomical Automatic Labeling (AAL) and associated intrinsic networks related to the 25 FCs. The width of the line represents the effect size of the difference (*t*-value) in the FC values between MDD and HC groups. DMN: default mode network; FPN: fronto-parietal network. An interactive display of each connection in Fig. 4a can be accessed from <https://bicr.atr.jp/~imamizu/p5/MDD/>.

**Response to Reviewer #2:**

We appreciate the reviewer’s comments. We have taken all the comments into account. Our responses to the reviewer’s comments are below.

***Reviewer’s comment 1:*** *“First, though they started the paper with explaining the RDoC initiative, they still treat the depression as a single diagnostic entity. This lumping approach has been criticized since early 2000 (Hyman, 2008, Nature; Miller, 2010, Science) and the criticism regarding the lumping approach provided the biggest motivation for the RDoC initiative. Depression is a highly heterogeneous condition (Insel & Cuthbert, 2015, Science), and therefore with this type of simple MDD vs. HC classifier, it is difficult to know what the model really captures because the MDD as a whole can mean many different things. They suggested that their model captures the MDD-ness and also anhedonia that is common across MDD and SCZ, but it struck me that the generalization to SCZ not to ASD is not enough to justify this "anhedonia" interpretation because SCZ and MDD shares many features other than anhedonia (e.g., Borsboom & Cramer, 2013).”*

***Response:*** We entirely agree with the reviewer’s point. We have now added the reviewer’s points to the Introduction section. As the reviewer mentioned, since major depressive disorder (MDD) is a highly heterogeneous condition, our final goal is the redefinition and subtyping of psychiatric disorders, agnostic to diagnosis. Although our hierarchical supervised / unsupervised approach starts with supervised learning based on a diagnosis, the diagnosis is only used as a single piece of information to extract important functional connectivity for future subtyping thereafter. We then used the identified functional connections for biologically meaningful dimensions of the disorder and applied unsupervised learning to these low biological dimensions to further understand psychiatric disorders (redefinition and subtyping). To achieve the final goal, it is an absolutely necessary prerequisite to first treat MDD as a single entity using diagnoses as supervised data and construct a brain network marker that generalizes to a completely independent data collected from multiple imaging sites. We included the above explanation in the Introduction section with more clarity than before.

As the reviewer mentioned, a previous study suggests that not only anhedonia, but also negative symptoms such as apathy and lethargy, are common symptoms between MDD and schizophrenia (SCZ) (Barch, Pagliaccio, & Luking, 2016; Ferenczi et al., 2016; Hallford & Sharma, 2019; Kollias et al., 2008; Marder & Galderisi, 2017; Spano et al., 2019). Thus, the Discussion section has been revised to mention only negative symptoms in SCZ and not anhedonia in the context of common symptoms between MDD and SCZ.

***Changes:*** We have revised the Introduction section of the manuscript, which now contains the following paragraph and sentences: “We believe that diagnosis of MDD based on Diagnostic and Statistical Manual of Mental Disorders (DSM) is not easy…” (page 7 line 146– page 8 line 155), “Major depressive disorder (MDD) is a highly heterogeneous psychiatric disorder, …” (page 4 lines 55–75), “Our purpose at this stage is to find …” (page 5 lines 80–83), “a lumping category such as a diagnosis,…” (page 5 lines 90–91), and “To achieve the final goal (redefinition and subtyping),…” (page 6 lines 105–108).

We have deleted the speculative discussions with regard to the "anhedonia" interpretation. We have revised the Discussion section of the manuscript, which now simply contains the following sentence: “Considering that the negative symptoms of schizophrenia are similar those of depression,…” (page 23 line 515–page 24 line 519).

**References:**

Barch, D. M., Pagliaccio, D., & Luking, K. (2016). Mechanisms Underlying Motivational Deficits in Psychopathology: Similarities and Differences in Depression and Schizophrenia. Curr Top Behav Neurosci, 27, 411-449. doi:10.1007/7854_2015_376

Ferenczi, E. A., Zalocusky, K. A., Liston, C., Grosenick, L., Warden, M. R., Amatya, D., . . . Deisseroth, K. (2016). Prefrontal cortical regulation of brainwide circuit dynamics and reward-related behavior. Science, 351(6268), aac9698. doi:10.1126/science.aac9698

Hallford, D. J., & Sharma, M. K. (2019). Anticipatory pleasure for future experiences in schizophrenia spectrum disorders and major depression: A systematic review and meta-analysis. Br J Clin Psychol, 58(4), 357-383. doi:10.1111/bjc.12218

Kollias, C. T., Kontaxakis, V. P., Havaki-Kontaxaki, B. J., Stamouli, S., Margariti, M., & Petridou, E. (2008). Association of physical and social anhedonia with depression in the acute phase of schizophrenia. Psychopathology, 41(6), 365-370. doi:10.1159/000152378

Marder, S. R., & Galderisi, S. (2017). The current conceptualization of negative symptoms in schizophrenia. World Psychiatry, 16(1), 14-24.

Spano, M. C., Lorusso, M., Pettorruso, M., Zoratto, F., Di Giuda, D., Martinotti, G., & di Giannantonio, M. (2019). Anhedonia across borders: Transdiagnostic relevance of reward dysfunction for noninvasive brain stimulation endophenotypes. CNS Neurosci Ther. doi:10.1111/cns.13230

***Reviewer’s comment 2:*** *“Second, in the previous revision, the classification performance for ASD was also significant (p = 2.0 * 10^-7; this is the quote from the previous manuscript, "The separation between ASD individuals and their healthy controls was poorer than that of SCZ but statistically significant (Fig. 8c; AUC = 0.57). "), and I wonder what has been changed since then. One change I noticed that in the previous paper, they reported the AUC, but in the current manuscript, they reported only the accuracy. Considering that this change (sig -> non-sig for ASD classification) seems important for the functional interpretation of the model, the authors should clarify what has been changed and whether the changes can be justified.”*

***Response:*** In our previous manuscript, we tested significance of the differences in the distributions of probability of depression between autism spectrum disorder (ASD) and healthy control groups in the independent validation dataset. However, to investigate the association with MDD in ASD, we rethought that it would be more important to investigate the probability that patients with ASD are diagnosed with MDD. To this end, in this manuscript, we tested whether patients with ASD were significantly diagnosed with MDD. As a result, patients with ASD were not significantly classified as having MDD (accuracy = 55%, *p* = 0.20, a two-way binomial test). Based on this result, we concluded that patients with ASD are unlikely to tend to have MDD.

**Response to Reviewer #3:**

We appreciate the reviewer’s critical comments and helpful suggestions. We have taken all the comments and suggestions into account, which helped us to improve our manuscript. Our responses to the reviewer’s comments are below.

***Reviewer’s comment 1:*** *“1) The paper extensively discusses the utility harmonizing rating state data across different centers. The effect size of this procedure remains unclear and needs to be demonstrated. There is a second worry along this line. The harmonization correction used for discovery and validation data were not the same. How this difference effects outcomes need also further clarification.”*

***Response:*** Following the reviewer’s comments, to demonstrate the utility of harmonization, we investigated whether the prediction performance and the number of selected functional connections (FCs) are different dependent on three harmonization schemes (without any harmonization, with ComBat harmonization, or with traveling subject (TS) harmonization) for the discovery dataset and two schemes (without any harmonization or with ComBat harmonization) for the independent validation dataset, respectively. We assessed the number of selected FCs, because we hypothesized that harmonization could improve not only the prediction performance but also the information (on important connections for the prediction) that we can extract from the dataset. In the end, we constructed three brain network markers dependent on three harmonization schemes for the discovery dataset and tested their prediction performances in the independent validation dataset with two harmonization schemes. We tested our hypothesis that harmonization improves the degree of separation of the depression probability distributions for healthy control (HC) and major depressive disorder (MDD) groups. A statistic was calculated by *p*(MDD) – 0.5 for MDD and 0.5 – *p*(MDD) for HC. A paired *t*-test was performed to compare the statistics of the individual classifiers. As a result, we were able to find a significant improvement in the prediction performance by harmonization for the independent validation dataset (Without vs ComBat: *t_439_* = 4.41, *p* = $1.3\times{10}^{-5}$). In addition, when we estimated the cutoff value for the probability of the MDD distribution in the data from OpenNeuro (OTHER), the cutoff value became more plausible (0.59🡪0.54) after harmonization in conjunction with the independent validation dataset (S2 Fig.). In contrast, we found no significant improvement in the prediction performance by harmonization for the discovery dataset, finding significant depreciation instead (Without vs ComBat (0.77🡪0.78): *t_439_* = 0.47, *p* = 0.64), Without vs TS (0.77🡪0.76): *t_439_* = -2.50, *p* = 0.013). Although a decrement of the AUC value was only 0.01, this result indicates that TS harmonization may worsen the prediction performance. This may be due to the difference in the harmonization method between the discovery dataset (TS harmonization) and the validation dataset (ComBat harmonization). The reason for which the harmonization for the discovery dataset did not improve the prediction performance may be due to the fact that the discovery dataset was acquired using a unified imaging protocol. Thus, the effect of harmonization might be small. On the other hand, the number of selected FCs was largest for TS harmonization (25FCs), compared to 23 and 21 for without harmonization and ComBat, respectively. This result indicates that we could extract more information about MDD from the data by TS harmonization method. We explained these results in the Discussion section, and added the S3 Text, S2 Figure, and S6 Table to the supplementary information.

***Changes:*** We have revised the Results section of the manuscript, which now contains the following paragraph: “To assess the effects of harmonization,…” (page 17 line 363–page 18 line 378).

| Supplementary table 6: Prediction performances in the independent validation dataset | | | | |
| --- | --- | --- | --- | --- |
|  | | *Harmonization for the discovery dataset* | | |
|  |  | Without harmonization | ComBat | Traveling subject |
| Number of connections | | 23 connections | 21 connections | 25 connections |
| *Harmonization for the independent validation dataset* | Without harmonization | AUC = 0.74  Accuracy = 0.68  Sensitivity = 0.72  Specificity = 0.65 | AUC = 0.74  Accuracy = 0.69  Sensitivity = 0.74  Specificity = 0.65 | AUC = 0.73  Accuracy = 0.67  Sensitivity = 0.71  Specificity = 0.63 |
|  | ComBat | AUC = 0.75  Accuracy = 0.67  Sensitivity = 0.74  Specificity = 0.61 | AUC = 0.75  Accuracy = 0.68  Sensitivity = 0.74  Specificity = 0.62 | AUC = 0.74  Accuracy = 0.66  Sensitivity = 0.72  Specificity = 0.61 |

**S2 Fig. Comparing the prediction performances among the harmonization schemes.** (**a**) The prediction performance (AUC) of the major depressive disorder (MDD) classifier in the independent validation dataset for each harmonization scheme for the discovery dataset and the independent validation dataset (without harmonization; blue bar, ComBat harmonization; yellow bar). (**b**) Probability distributions for the diagnosis of MDD in the data from OpenNeuro (OTHER) without harmonization or with ComBat harmonization. AUC: Area under the curve, MCC: Matthews correlation coefficient.

***Reviewer’s comments 2:*** *“2) There is no evidence that the authors corrected for age and gender influences on FC values. Both of these are well known modulators of FC and the various data sets used in the study are slightly different on these parameters. One wonders if predictability would improve once such influences are corrected for. It is also likely that age and gender themselves influence on head motion. At least the extent of head motion contribution to predictability needs to be identified, with/without age gender effects.” a*nd *“5) Head motion remains a main worry in constructing FC matrices. It would be nice to show that resultant matrices were NOT or minimally affected by head motion.”*

***Response:*** We agree with the reviewer’s comment that it is important to rule out the possibility that the classifier’s performance is driven by confounds. Therefore, we tested whether we could predict classifier scores (probability of depression) from age, sex, an amount of motion (frame-wise displacement [FD]), or the combination of head movement parameters (x, y, z, yaw, pitch, and roll) (Kohoutova et al., 2020), respectively. We evaluated the performances by 10-fold cross-validation in the discovery dataset. As a result, only age could significantly predict the classifier scores (age: *r* = 0.26, *p* < 0.0001, sex: *r* = 0.069, *p* > 0.07, FD: *r* = -0.031, *p* > 0.42, motion parameters: *r* = 0.06, *p* > 0.09). This result indicates that the classifier’s performance was influenced by age, but not sex, the amount of motion, or the combination of head movement parameters.

Given the above, to remove the age effect on the classifier’s scores, we changed the method for constructing a classifier. When we performed an under-sampling and subsampling procedure, we matched the mean age between major depressive disorder (MDD) and healthy control (HC) groups in each subsample. As a result, the area under the curve (AUC), accuracy, sensitivity, and specificity were 0.74, 66%, 72%, and 65%, respectively, in the discovery dataset. In the validation dataset, AUC, accuracy, sensitivity, and specificity were 0.76, 68%, 70%, and 66%, respectively. These results indicate that the classifier performances did not change even after changing the method. Next, we tested whether we could predict classifier scores based on age by a 10-fold cross-validation. Although only age could predict the classifier scores (age: *r* = 0.14, *p* < 0.0005, sex: *r* = 0.029, *p* > 0.45, FD: *r* = 0.07, *p* > 0.05, motion parameters: *r* = 0.038, *p* > 0.32), the effect became smaller. This result raises two possibilities, 1) the classifier outputs were really affected by age, or 2) difference in mean age between MDD and HC groups (mean age: MDD = 41.8, HC = 37.4) led to the pseudo correlation between age and probability of depression. To test the latter possibility, we prepared 10 subsample datasets in which the mean age was matched between MDD and HC groups from the discovery dataset (differences in mean age between MDD and HC in 10 subsamples: *t*-value = -0.021±0.057 [mean ±1 SD]). MDD data was the same but HC data was different among the 10 subsamples. If the latter is correct, we cannot predict the classifier scores from age. As a result, we could not significantly predict the classifier scores in any of the subsamples (*r* = 0.0195±0.0677 [mean ± 1SD], *p* = 0.06~0.63). This result suggests that the difference in mean age between MDD and HC groups led to the pseudo correlation. In other words, the classifier's performance was unlikely driven by age. We included all above analyses in the Results section and modified the Methods and Results section to update the method for constructing a classifier, classification performances, and the important functional connections for MDD diagnosis.

Furthermore, the most reliable way to confirm that the classifier's performance is not driven by age is to test if we could obtain an adequate generalization performance in age-controlled datasets. Therefore, we prepared 1,000 age matched subsamples from the validation dataset (differences in mean age between MDD and HC in the 1,000 subsamples: *t*-value = 0.0052± 0.042 [mean ± 1SD], S1 Fig.). MDD data was the same but HC data was different among the 1,000 subsamples. The generalization performances were almost the same as that using the whole validation dataset (AUC = 0.756 ± 0.0076 [mean ± 1SD], S1 Fig.). In addition to this result, our new result that showed our success of generalization (AUC = 0.68) to the dataset collected from a country outside Japan (OpenNeuro: <https://openneuro.org/datasets/ds002748/versions/1.0.0>) also ruled out the possibility that the classifier’s performance was driven by age, because mean age between MDD and HC groups were matched in this dataset (MDD = 32.7 ± 8.94 [mean ± 1SD], HC = 33.8 ± 8.49 [mean ± 1SD], *t*-test: *t*_68_ = -0.5, *p* = 0.62) (see detail in our response to the reviewer 3’s comment 3). We added this analysis to the S2 Text and S1 Figure in the supplementary information.

***Changes:*** Following the change in the method to construct a brain network marker, we changed all main results corresponding to the change in the method (prediction performances and selected functional connections).

We have revised the Results section of the manuscript, which now contains the following paragraph: “To rule out the possibility that the classifier’s performance is…” (page 15 line 313–page 18 line 320).

**References:**

Kohoutova, L., Heo, J., Cha, S., Lee, S., Moon, T., Wager, T. D., & Woo, C. W. (2020). Toward a unified framework for interpreting machine-learning models in neuroimaging. Nat Protoc, 15(4), 1399-1435. doi:10.1038/s41596-019-0289-5

**S1 Fig. Distribution of prediction performance and difference in mean age between MDD and HC across all subsamples.** The distribution of area under the curve (AUC) and *t*-value (difference in mean age between major depressive disorder (MDD) and healthy control (HC) groups) across all subsamples.

***Reviewer’s comment 3:*** *“3) The discovery and validation approach is very good. However, it is all based on data collected in Japan. The results would be more convincing if they can generalize to data collected in other centers. Perhaps the authors can test some of the Drysdale data that is publicly available. This is specially important as the discussion mentions that some of the specific links are shared between them and Drysdale.”*

***Response:*** Although we searched Drysdale’s publicly available dataset including major depressive disorder (MDD) data following the reviewer’s suggestion, we were not able to find it (Drysdale’s paper said that some of control scans and a data of schizophrenia were used from publicly available dataset [1000 functional connectome project]). Instead, we found a publicly available resting-state fMRI dataset including MDD in OpenNeuro (21 healthy controls and 51 MDD patients: <https://openneuro.org/datasets/ds002748/versions/1.0.0>), and applied our brain network marker to this dataset. We preprocessed this dataset using the same procedure as in this paper. Harmonization was performed in conjunction with our independent validation dataset using ComBat. As a result, the area under the curve (AUC), accuracy, sensitivity, and specificity were 0.68, 63%, 69%, and 48%, respectively (R1 Fig. and Fig. 3d). Although specificity was low due to imperfect harmonization (It can be seen from the deviation of the threshold from 0.5 in R1 Fig. and Fig. 3d), this result indicates that our brain network marker could be generalized to the dataset acquired from within a country other than Japan. We added this result to the Results section and revised all related parts.

**R1 Fig. Probability distribution for the diagnosis of MDD in the data from OpenNeuro (OTHER).** The probability distribution for the diagnosis of major depressive disorder (MDD) in the data from OpenNeuro (OTHER). MDD and healthy control (HC) distributions are depicted in red and blue, respectively. AUC: Area under the curve, MCC: Matthews correlation coefficient.

***Reviewer’s comment 4:*** *“4) The parcellation scheme used seems unique to the group. It would be nice if higher/lower resolution parcellations can also be tested. This would address the extent to which observed results are robust and independent of the specifics of parcellation.”*

***Response:*** We thank the reviewer very much for this suggestion. We investigated the prediction performance when we used a parcellation scheme other than Glasser’s region of interest (ROI). We investigated Shen’s ROI (Shen, Tokoglu, Papademetris, & Constable, 2013) and Power’s ROI (Power et al., 2011) which are well used in the previous studies (Drysdale et al., 2017, Rosenberg et al., 2016). As a result, the area under the curve (AUC) in the independent validation dataset was 0.74 for Shen’s ROI and 0.76 for Power’s ROI, respectively. This result indicates that there was no difference compared with the prediction performance using Glasser’s ROI (AUC=0.74).

Furthermore, we investigated the effect of higher/lower resolution in the parcellation on prediction performance by using Schaefer’s ROI (Schaefer et al., 2018). Since there are from 100 to 1,000 ROIs in increments of 100, Schaefer’s ROI is suitable for investigating the effect of higher/lower resolution parcellations. We excluded 1,000 ROI from this analysis, because the scanned volume did not cover the all 1,000 ROIs. As a result, the max AUC was achieved when we used 400 ROIs (AUC = 0.75). The AUC increased monotonically up to 400 ROIs, and decreased over 400 ROIs (S4 Fig.). The accuracy as a function of the number of ROIs showed a similar profile to the AUC, but the maximum accuracy was achieved when we used 500 ROIs (accuracy = 69%). Thus, our choice of Glasser’s 379 ROIs was fair enough. We discussed these results in the Discussion section and added the S5 Text and S4 Figure to the supplementary information.

***Changes:*** We have revised the Results section of the manuscript, which now contains the following sentences: “We further assessed the prediction performance…” (page 18 lines 385–388).

***References:***

Power, J. D., Cohen, A. L., Nelson, S. M., Wig, G. S., Barnes, K. A., Church, J. A., . . . Petersen, S. E. (2011). Functional network organization of the human brain. Neuron, 72(4), 665-678. doi:10.1016/j.neuron.2011.09.006

Shen, X., Tokoglu, F., Papademetris, X., & Constable, R. T. (2013). Groupwise whole-brain parcellation from resting-state fMRI data for network node identification. NeuroImage, 82, 403-415. doi:10.1016/j.neuroimage.2013.05.081

Drysdale, A. T., Grosenick, L., Downar, J., Dunlop, K., Mansouri, F., Meng, Y., . . . Liston, C. (2017). Resting-state connectivity biomarkers define neurophysiological subtypes of depression. Nat Med, 23(1), 28-38. doi:10.1038/nm.4246

Rosenberg, M. D., Finn, E. S., Scheinost, D., Papademetris, X., Shen, X., Constable, R. T., & Chun, M. M. (2016). A neuromarker of sustained attention from whole-brain functional connectivity. Nat Neurosci, 19(1), 165-171. doi:10.1038/nn.4179

Schaefer, A., Kong, R., Gordon, E. M., Laumann, T. O., Zuo, X. N., Holmes, A. J., . . . Yeo, B. T. T. (2018). Local-Global Parcellation of the Human Cerebral Cortex from Intrinsic Functional Connectivity MRI. Cereb Cortex, 28(9), 3095-3114. doi:10.1093/cercor/bhx179

**S4 Fig. Higher/lower resolution region of interests.** The prediction performances (Area under the curve [AUC], accuracy, specificity, and sensitivity) of the major depressive disorder (MDD) classifier constructed by Schaefer’s regions of interest (ROIs) as a function of the number of ROIs.

***Reviewer’s comment 5:*** *“6) Although the authors claim to use independent discovery and validation data sets. It is not always clear that this separation was properly followed. Please indicate more clearly when it was or was not.”*

***Response:*** We thank the reviewer for pointing out this important issue. Within the Strategic Research Program for Brain Sciences (SRPBS), DecNef project started at 2013, we planned to acquire the rs-fMRI data using a unified imaging protocol (S1 Table; <http://bicr.atr.jp/rs-fmri-protocol-2/>). The discovery dataset was acquired in this project using the unified imaging protocol from 2014. In contrast, the independent validation dataset was acquired from other projects from 2008. The dataset collected from Hiroshima University Hospital (HUH), Hiroshima Kajikawa Hospital (HKH), and Hiroshima Rehabilitation Center (HRC) in the independent validation dataset was acquired by “Development of diagnosis and treatment techniques for patients with severe intractable depression and insensitivity to antidepressant treatment based on molecular and cellular researches on BDNF and depression“ of the Japan Science and Technology (JST) Agency Core Research for Evolutional Science and Technology (CREST) from 2008 and by “Understanding the neurocircuit - molecular mechanism underlying pathophysiology of depression and the development of its neuroscience-based diagnosis and treatment” of the SRPBS from 2011. The dataset collected from Yamaguchi University (UYA) was acquired by “Exploration of the biological markers for discrimination of heterogeneous pathophysiology of major depressive disorder” from the SRPBS from 2012. To clearly describe this difference between the datasets, we added a brief explanation in the Introduction section and added precise information to this effect in the Methods section.

***Changes:*** We have revised the Introduction section of the manuscript, which now contains the following sentences: “Specifically, for constructing a brain network marker, …” (page 6 line 119–page 7 line 132).

We have revised the Methods section of the manuscript (page 25 line 559–page 26 line 573).

***Reviewer’s comment 6:*** *“7) Obtained results would be further strengthened if identified links or some subset of them actually tracked the primary variable on which the data are being separated: BDI scale.”*

***Response:*** We thank the reviewer very much for this suggestion. We investigated the correlation between the classifier’s output (probability of depression) and depressive symptoms (Beck Depression Inventory-II score). We found significant correlations in both the discovery dataset and validation dataset (discovery dataset: *r* = 0.29, *p* < $1.0\times{10}^{-10}$, validation dataset: *r* = 0.32, *p* < $1.0\times{10}^{-11}$). However, we were not able to find consistently significant correlations within each group (healthy control (HC) group and major depressive disorder (MDD) group) in the both datasets (discovery dataset: MDD [*r* = -0.07, *p* = 0.46], HC [*r* = 0.13, *p* =0.010]; validation dataset: MDD [*r* = 0.0043, *p* =0.95], HC [*r* = -0.13, *p* = 0.04]). We included this result in the Results section.

***Changes:*** We have revised the Results section of the manuscript, which now contains the following paragraph: “Furthermore, we investigated the correlation between the classifier’s output…” (page 15 lines 292–298).
