# Supplementary material for: Generalizable brain network markers of major depressive disorder across multiple imaging sites
Source: PLoS Biol. 2020 Dec 7;18(12):e3000966. doi: 10.1371/journal.pbio.3000966 (PMC7721148; doi:10.1371/journal.pbio.3000966)
Supplement: Attachment — Submitted filename: ResponseLetter_PLOSBiology.docx [file pbio.3000966.s020.docx]

We had no comments from the reviewers but only from the editors.

Our responses to the editors’ comments are described in the cover letter.
